# Supplementary material for: Modelling Skylarks (Alauda arvensis) to Predict Impacts of Changes in Land Management and Policy: Development and Testing of an Agent-Based Model
Source: PLoS One. 2013 Jun 6;8(6):e65803. doi: 10.1371/journal.pone.0065803 (PMC3675089; doi:10.1371/journal.pone.0065803)
Supplement: Supporting Information S4 — The skylark ODdox as a zipped archive. (ZIP) [file pone.0065803.s004.zip › Skylark_ODdox/populationmanager_8h.html]

ALMaSS Skylark ODdox: populationmanager.h File Reference


ALMaSS Skylark ODdox
 2.0


- Main Page
- Related Pages
- Classes
- Files

- File List
- File Members

Classes |
Typedefs

populationmanager.h File Reference

**PopulationManager.h This is the header file for the population manager and associated classes**   
More...

|  |  |
| --- | --- |
| Classes | |
| class | AnimalPosition |
|  | A class defining an animals position. More... |
| class | APoint |
|  | A struct defining an x,y coordinate set. More... |
| struct | IntArray100 |
|  | A struct of 100 ints. More... |
| class | Population\_Manager |
|  | Base class for all population managers. More... |
| class | probe\_data |
|  | Data structure to hold & output probe data probe data is designed to be used to return the number of objects in a given area or areas in specific element or vegetation types or farms. More... |
| struct | rectangle |
|  | A struct defining two x,y coordinate sets of positive co-ords only. More... |
| class | RoeDeerInfo |
|  | Part of the basic ALMaSS system (obselete) More... |
| class | TALMaSSObject |
|  | The base class of all ALMaSS objects requiring Step code. More... |
| class | TAnimal |
|  | The base class for all ALMaSS animal classes. More... |

|  |  |
| --- | --- |
| Typedefs | |
| typedef char \* | AnsiString |
| typedef vector< TAnimal \* > | TListOfAnimals |

---

## Detailed Description

**PopulationManager.h This is the header file for the population manager and associated classes**

by Chris J. Topping   
Version of 23rd July 2003   
  
With additions as noted in:   
January 2008   
Doxygen formatted comments in May 2008

---

## Typedef Documentation

typedef char\* AnsiString

typedef vector< TAnimal \* > TListOfAnimals


- CJT
- MSVC
- ALMaSS Working Source
- BatchALMaSS
- populationmanager.h
- Generated on Thu Jan 10 2013 13:15:35 for ALMaSS Skylark ODdox by
   1.8.1.1
